# The efficacy and safety of massage adjuvant therapy in the treatment of diabetic peripheral neuropathy: A protocol for systematic review and meta-analysis of randomized controlled trials

**DOI:** 10.1097/MD.0000000000029032

**Published:** 2022-03-11

**Authors:** Longsheng Ren, Ruiying Guo, Guojing Fu, Jie Zhang, Qiang Wang

**Affiliations:** a Shandong Provincial Hospital Affiliated to Shandong First Medical University, Jinan, Shandong, China; b Shandong University of Traditional Chinese Medicine, Jinan, Shandong, China.

**Keywords:** diabetic peripheral neuropathy, massage, protocol, systematic review and meta-analysis

## Abstract

**Background:**

The incidence of diabetic peripheral neuropathy (DPN) is increasing year by year. If patients cannot receive timely and effective treatment, DPN may lead to diabetic foot ulcers or even amputation. This risk factor has been widely concerned around the world. Massage, as a non-invasive physical therapy method, is gradually being applied in the adjuvant treatment of DPN. However, there is no systematic review of the adjuvant treatment of DPN by massage. Our study will explore the effectiveness and safety of massage applied in DPN.

**Methods:**

Eight electronic databases (PubMed, Cochrane, Web of Science, Sinomed, Embase, China National Knowledge Infrastructure, WanFang Data, Chongqing VIP Information) will be searched by our computer on February 9, 2022. A randomized controlled trial (RCT) of adjuvant massage therapy for DPN was screened. Primary outcome measures: efficiency, nerve conduction velocity. Secondary outcome measures: pain, blood glucose, and incidence of adverse reactions. The quality of the study was evaluated by two researchers using the RCT bias risk assessment tool in the Cochrane review manual Handbook5.4, and meta-analysis was performed by RevMan5.4 software.

**Results:**

RCTs will be used to evaluate the clinical efficacy of massage adjuvant therapy in DPN.

**Conclusion:**

This study will provide evidence-based evidence for the safety and effectiveness of massage adjuvant therapy in DPN.

**Protocol registration number:**

INPLASY202220025.

## 1 Introduction

Diabetic peripheral neuropathy (DPN) is a common and serious chronic complication in diabetic patients.^[1]^ The incidence of DPN is also increasing due to the expansion of the diabetic population. Studies show that the incidence of DPN is 7% in the world.^[2,3]^ The main symptoms of this disease are symmetrical limb numbness, pain, muscle atrophy and other clinical manifestations.^[4]^ DPN often affects the terminal limbs of patients, and in severe cases will lead to amputation of patients, which will greatly affect the quality of life of patients and the social medical environment.^[5,6]^ The pathogenesis of DPN is still unclear, and modern medicine believes that it may be related to the impairment of blood vessel and nerve function in patients with diabetes.^[7]^ Studies have shown that when blood glucose is kept at a high level, a series of reactions such as metabolic disorders, vascular dysfunction, lack of neurotrophic factors, and local oxidative stress will occur in patients, which will eventually lead to neuropathy.^[8]^ If DPN is not treated promptly and effectively, it can lead to secondary local infections, ulcers, and deep tissue destruction, ultimately resulting in disability. At present, the main treatment for DPN is to use medication according to different symptoms of patients on the premise of controlling blood glucose, such as using neurotrophic drugs to promote nerve repair, using antidepressants to relieve patients’ anxiety, using opioids to relieve patients’ pain. However, long-term drug use may lead to constipation, mood disorders, fatigue and other side effects, and the quality of life of patients will be seriously affected.^[4,9,10]^ Therefore, seeking a safe and effective method to treat DPN has become an urgent problem to be solved.

Massage can play a role in improving local microcirculation, increasing blood flow rate and correcting metabolic disorders.^[11]^ In recent years, studies have found that the application of massage in DPN can improve the blood circulation of local tissues, improve the body's local pain threshold, enhance the range of motion of joints and improve the blood supply and nutrition metabolism of peripheral nerves.^[1]^ However, there is currently no meta-analysis on the efficacy of massage in the treatment of DPN. Our study will be based on a RCT, using meta-analysis to evaluate the efficacy and safety of massage in the treatment of DPN, so as to enrich the clinical treatment methods of DPN.

## 2 Methods

### 2.1 Study registration

Our protocol has been registered on the INPLASY website (https://inplasy.com/) under the registration number INPLASY202220025.

### 2.2 Ethics and dissemination

The studies we selected were published RCTs, so we did not need to obtain ethical approval and informed consent from patients. We upload our findings to peer-reviewed journals.

### 2.3 Inclusion criteria for this study

#### 2.3.1 Types of studies

All the studies we selected were RCTS of massage therapy for DPN. Studies that are not RCTS will be excluded: for example, animal studies, case reports, reviews, and basic studies.

#### 2.3.2 Types of participants

Regardless of race, nationality, age or gender, patients who meet the diagnostic criteria for DPN will be included in our study.

#### 2.3.3 Types of interventions

The control group was given hypoglycemic treatment and symptomatic treatment, and the treatment group was combined with massage treatment on the basis of the control group. And the massage method chosen may vary from one RCT to another.

#### 2.3.4 Types of outcome measures

Primary outcome measure: efficiency, nerve conduction velocity.

Secondary outcome measures: pain, blood glucose, incidence of adverse reactions.

### 2.4 Data sources

#### 2.4.1 Electronic searches

The documents we need are retrieved from 8 electronic databases (PubMed, Cochrane, Web of Science, Sinomed, Embase, China National Knowledge Infrastructure, WanFang Data, Chongqing VIP Information) by computer. The deadline is February 9, 2021. In The Chinese database, the main search terms are extended according to the requirements, while in the English database, all the literatures in each database are retrieved by MeSH subject terms plus free words. The retrieval method takes PubMed as an example, as shown in Table 1.

**Table 1:** Search strategy of PubMed.

Number search item	
#1	Diabetic peripheral neuropathy [MESH]
#2	Diabetic peripheral neuropathy [Title/Abstract] OR Diabetic Neuropathy [Title/Abstract] OR Neuropathies, Diabetic [Title/Abstract] OR Neuropathy, Diabetic [Title/Abstract] OR Diabetic Neuropathy, Painful [Title/Abstract] OR Diabetic Polyneuropathy [Title/Abstract] OR Foot, Diabetic [Title/Abstract]
#3	# 1 OR #2
#4	Massage [MESH]
#5	Massage [Title/Abstract] OR Zone Therapy [Title/Abstract] OR Zone Therapies [Title/Abstract] OR Therapy, Zone [Title/Abstract] OR Therapies, Zone [Title/Abstract] OR Massage Therapy [Title/Abstract] OR Massage Therapies [Title/Abstract] OR Therapy, Massage [Title/Abstract] OR Therapies, Massage [Title/Abstract] OR Tuina [Title/Abstract] OR Japanese shiatsu [Title/Abstract] OR Thai massage [Title/Abstract] OR Swedish massage [Title/Abstract]
#6	#4 OR #5
#7	Randomized controlled trial [Publication Type] OR randomized [Title/Abstract] OR randomly [Title/Abstract]
#8	#3 AND #6 AND #7

#### 2.4.2 Searching for other resources

We will manually search for books, potential gray literature, conference papers, and other RCTS involving massage therapy for DPN.

### 2.5 Data collection and analysis

#### 2.5.1 Selection of studies

**Figure 1:**
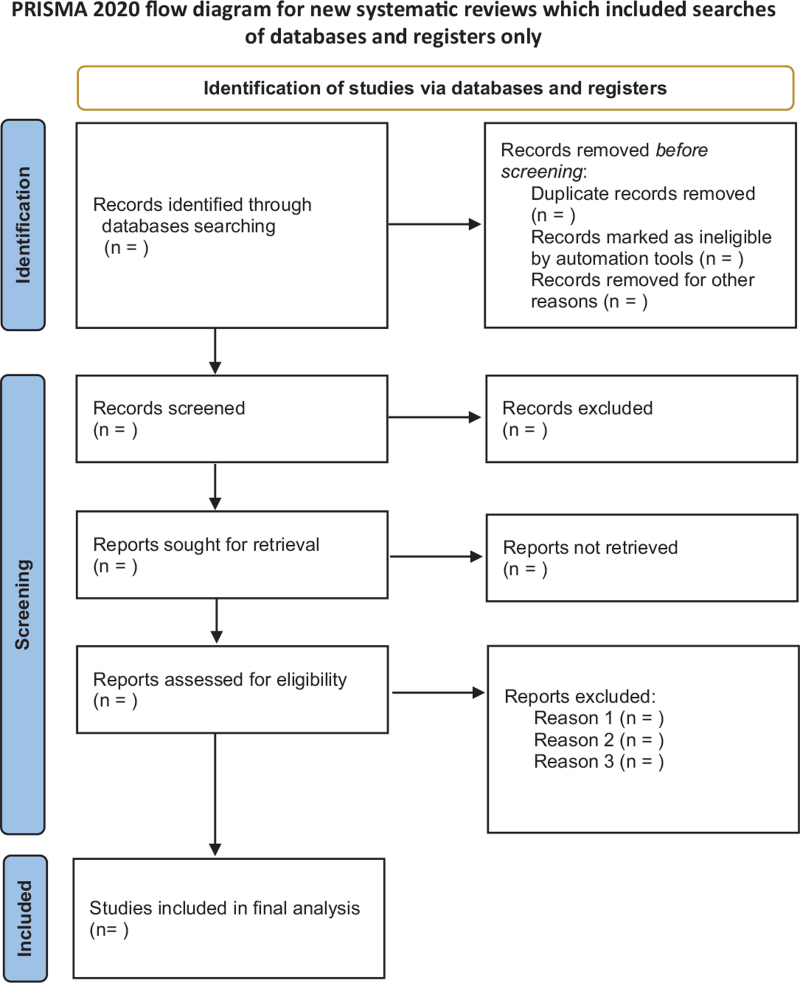
The flowchart of the screening process.

After the database is retrieved, the retrieved literatures are imported into EndNote X9 software for screening. After excluding the same literature, the two researchers (Longsheng Ren and Ruiying Guo) further read the title and abstract of the literature to exclude the literature that did not meet the requirements. Read the full text further to determine final inclusion. Disagreements arising in the process will be handed over to a third party (Guojing Fu). The process of literature screening is shown in Figure 1.

#### 2.5.2 Data extraction and management

The two researchers (Longsheng Ren and Ruiying Guo) will independently extract data information and compile the collected data information into Excel tables. The extracted information was as follows: publication year, first author, gender, age, course of disease, course of treatment, intervention measures, outcome indicators, sample size, adverse reactions, etc. If there is any disagreement, it will be decided by a third party (Guojing Fu). If the selected study has incomplete data, we will attempt to complete the information by contacting the original author, otherwise the study will be excluded.

#### 2.5.3 Risk of bias assessment

Two researchers (Longsheng Ren and Ruiying Guo) evaluated the quality of the included studies according to the Randomised controlled trial bias risk Assessment Tool (Cochrane Evaluation Handbook 5.4). The main evaluation items include: “random sequence generation,” “selective reporting,” “blindness,” “distribution concealment,” “incomplete result data,” etc. Each assessment item is classified as “low risk,” “unclear risk,” and “high risk.” If there is any disagreement, we will discuss with a third party (Guojing Fu) to resolve it.

#### 2.5.4 Data analysis

Data processing software RevMan 5.4 will be used for meta-analysis of the data included in the study. Binary data calculated the relative risk (RR) of 95% confidence intervals (CI), and continuous data calculated the mean difference (MD) of 95% CI. *P* < .05 was used as the standard for statistically significant differences. *P* and *I*^2^ values were used to test the heterogeneity of the included literature. *I*^2^ < 50% was considered as the standard with no heterogeneity or low heterogeneity, and fixed effect model was used for analysis. *I*^2^ > 50% was considered as the criterion of existence or large heterogeneity, and random effect model was used for analysis. The source of heterogeneity will be determined by sensitivity analysis or subgroup analysis, and we will use descriptive analysis if meta-analysis cannot be carried out smoothly.

#### 2.5.5 Subgroup analysis

Subgroup analysis was performed based on age, course of disease, duration of treatment, and different interventions to explore the sources of heterogeneity.

#### 2.5.6 Sensitivity analysis

The source of heterogeneity present in the study will be determined by sensitivity analysis.

#### 2.5.7 Assessment of reporting biases

If more than 10 studies are included, a funnel plot will be drawn to analyze whether there is publication bias in this study, and the results will be analyzed and explained. Funnel plots will be drawn using Revman5.4 software.

#### 2.5.8 Grading the quality of evidence

In this study, we will use internationally recognized recommendation rating methods to evaluate the quality of outcome evidence. The quality of evidence is classified as “very low quality,” “low quality,” “medium quality,” and “high quality.”

## 3 Discussion

The incidence of DPN is closely related to diabetes. Studies have found that diabetics have up to a 50% chance of developing peripheral neuropathy.^[12,13]^ DPN, if not treated promptly and effectively, is an important risk factor for diabetic foot ulcers, with a serious risk of amputation and death.^[14,15]^ The persistence of DPN affects patients’ life, mood and sleep, and will further lead to patients’ pain, anxiety and depression, which has a great impact on patients’ body and mind.^[16]^ The treatment of DPN in modern medicine is usually based on blood glucose control, combined with the use of drugs to relieve other accompanying symptoms, with limited therapeutic effects and large side effects. In order to make up for the lack of therapeutic methods for DPN, reduce the adverse reactions caused by long-term drug use, and improve the quality of life of patients with DPN, we believe that massage adjuvant therapy can be used in the treatment of DPN. Studies have found that massage can promote the degradation and excretion of harmful substances in local tissues and peripheral blood, and play an important role in promoting metabolism and accelerating material exchange.^[17,18]^ In addition, massage can also improve the blood oxygen concentration of limb tissue, especially around nerve tissue, so it can further improve the nutritional state of nerve tissue and promote the repair of nerve cells.^[19,20]^ At the same time, massage can also reduce the pain of patients by improving the pain threshold of local tissues, and enhance the range of motion of patients’ limbs.^[21]^ Moreover, massage is a physical therapy method, which can effectively reduce the adverse reactions caused by the long-term use of drugs, and gradually get social recognition in the adjuvant treatment of DPN.^[22]^

However, massage therapy for DPN lacks evidence-based support, so we will conduct a systematic evaluation and meta-analysis on the efficacy and safety of massage adjuvant therapy for DPN, aiming to provide a more powerful clinical basis for massage therapy for DPN.

## Author contributions

**Conceptualization**: Longsheng Ren, Ruiying Guo, Qiang Wang.

**Data curation**: Longsheng Ren, Ruiying Guo.

**Formal analysis**: Longsheng Ren, Ruiying Guo.

**Funding acquisition**: Qiang Wang.

**Investigation**: Longsheng Ren, Ruiying Guo, Guojing Fu.

**Methodology**: Longsheng Ren, Ruiying Guo.

**Project administration**: Longsheng Ren, Ruiying Guo, Qiang Wang.

**Resources**: Longsheng Ren, Ruiying Guo.

**Software**: Longsheng Ren, Ruiying Guo, Jie Zhang.

**Supervision**: Qiang Wang.

**Validation**: Qiang Wang, Jie Zhang.

**Visualization**: Longsheng Ren, Ruiying Guo.

**Writing** – **original draft**: Longsheng Ren, Ruiying Guo, Qiang Wang.

**Writing** – **review** & **editing**: Longsheng Ren, Ruiying Guo, Qiang Wang.
